# Prediction of Selected Physical and Mechanical Properties of a Telechelic Polybenzoxazine by Molecular Simulation

**DOI:** 10.1371/journal.pone.0061179

**Published:** 2013-04-08

**Authors:** Wan Aminah Wan Hassan, Ian Hamerton, Brendan J. Howlin

**Affiliations:** Department of Chemistry, University of Surrey, Guildford, Surrey, United Kingdom; Bioinformatics Institute, Singapore

## Abstract

Molecular simulation is becoming an important tool for both understanding polymeric structures and predicting their physical and mechanical properties. In this study, temperature ramped molecular dynamics simulations are used to predict two physical properties (i.e., glass transition temperature and thermal degradation temperature) of a previously synthesised and published telechelic benzoxazine. Plots of simulated density *versus* temperature show decreases in density within the same temperature range as experimental values for the thermal degradation. The predicted value for the thermal degradation temperature for the cured polybenzoxazine based on the telechelic polyetherketone (PEK) monomer was *ca.* 400°C, in line with the experimental thermal degradation temperature range of 450°C to 500°C. Mechanical Properties of both the unmodified PEK and the telechelic benzoxazines are simulated and compared to experimental values (where available). The introduction of the benoxazine moieties are predicted to increase the elastic moduli in line with the increase of crosslinking in the system.

## Introduction

From the appearance of the first papers on Quantitative Structure Activity Relationships (QSAR) some 50 years ago [1], and Molecular Dynamics (MD) simulations a decade later, [2] the application of computational techniques to simulate or predict chemical properties has grown considerably in importance to become a routine method in the pharmaceutical industry in the search for new lead compounds for drug development. The materials industry has been somewhat more conservative in its slower adoption of the same techniques despite the undoubted power of the approach, although the more widespread availability of several commercial polymer-modelling programs makes it possible to incorporate these methods in the selection for new candidate polymers for specific applications based on *e.g.* their physical or mechanical properties.

**Figure 1:**
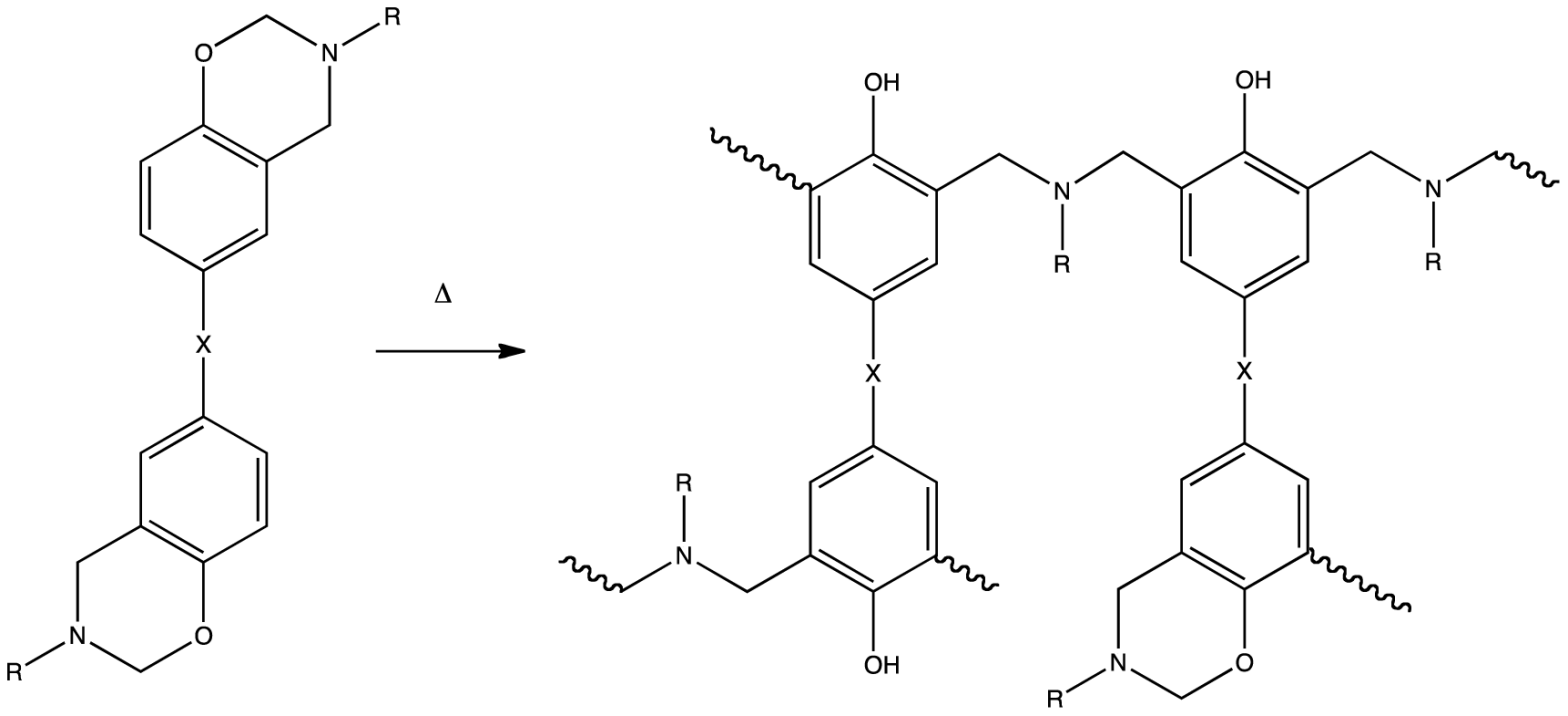
Polymerisation of bisbenzoxazines through ring opening and crosslinking, in this case *R* = methyl, *X* = the arylate backbone shown.

The computational techniques used within this work are MD simulations, which simulate a collection of atoms over a period of time. The interactions between the atoms in the MD simulations are based on Newton's laws of motion [3]. Using these classical mechanics to simulate the movement of the polymers under investigation, several physical properties of the polymer can be predicted. These include the volume, density, *T*_g_ and Young's modulus, as well as electronic conduction, blend miscibility and thermal stability of polymers [4]. The aim of this paper is to predict selected physical properties of polybenzoxazines using MD simulations. The basis of this paper is the replication of empirical data previously published by Nakamura and Ishida from research carried out at Case Western Reserve University (CWRU) [5] on a series of novel telechelic polybenzoxazines. The preparation of polymers of aromatic oxazines, or benzoxazines, date back some sixty years [6]; although the seminal work on the difunctional monomers is now some 18 years old [7]. Commercial exploitation of polybenzoxazines materials has only come about relatively recently and they are now receiving a great deal of academic and industrial interest [8]. Poly(*bis*-benzoxazine)s (sometimes simply referred to as polybenzoxazines) are a family of thermosetting polymers that are made up through step growth ring-opening polyaddition from *bis*-benzoxazine monomers (Figure 1), which are in turn the products of the Mannich reaction between a *bis*-phenol, formaldehyde and a primary amine [9].

**Figure 2:**
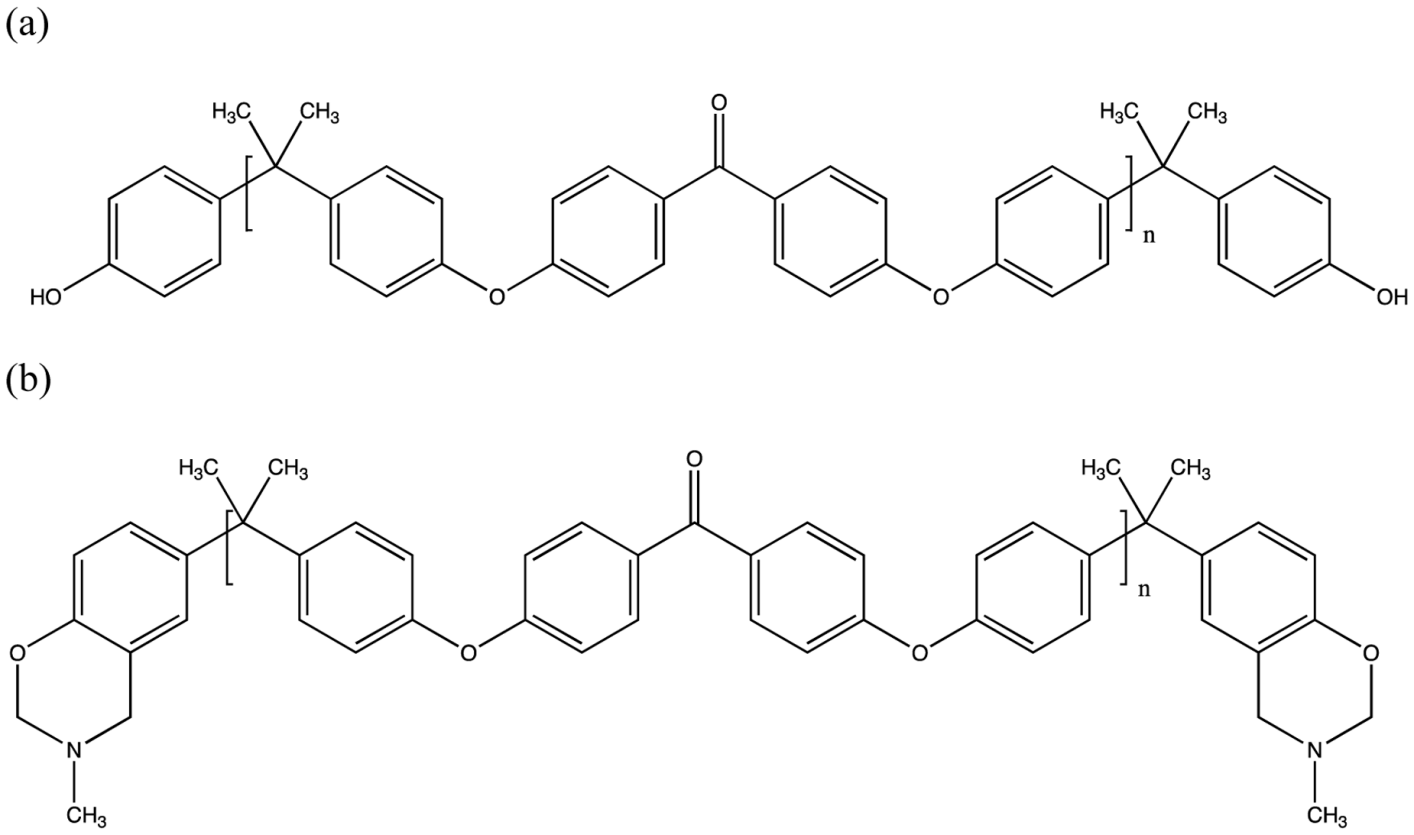
Structure of (a) the PEK oligomer precursor, *n* = 5 and (b) the telechelic *bis*-benzoxazine monomer, *n* = 5.

Polybenzoxazines appear to incorporate the best properties from conventional phenolics, and may find application in a number of their traditional niches, whilst improving on shelf life and offering the potential for greater toughness properties through their greater molecular flexibility. However, the relatively low fracture toughness that is achieved by cured polymers is still a problem in advanced aerospace applications when compared with competitor resins (a K_IC_ value of *ca.* 0.51–0.54 MPa.m^0.5^ is typical [10], although more recent experimental linear polymers achieve higher values). Consequently, one approach that has been taken to address this issue is through the examination of telechelic monomers (oligomers or prepolymers capable for further reaction through functional groups at the chain ends). The Telechelic monomers chosen are those in which the extended backbone would lead to a lower crosslink density (leading to a reduction in brittleness), whilst maintaining both thermal stability and glass transition temperature, *T*_g_, through the use of aryl moieties. This is an approach that has been previously employed with older thermosetting polymers such as bismaleimides and polyaspartimides [11] and polycyanurates [12]. In the original empirical study at CWRU a series of telechelic monomers based on a thermoplastic PEK (Figure 2a) were produced with various backbone chain lengths (from 1520–2940 Da), from which we selected 2200 Da as a suitable representative model. Gel permeation chromatography (GPC) and ^1^H nuclear magnetic resonance (NMR) spectroscopy were employed to determine the molecular weights of the species before thermal (differential scanning calorimetry, DSC, thermogravimetric analysis, TGA), thermomechanical (dynamic mechanical thermal analysis, DMTA) and ultimately mechanical testing were used to yield a variety of physical properties. From this series, a representative sample of 2200 Da (n = 5, derived from bisphenol A and 4,4′-dichlorobenzophenone, in the molar ratio 3∶2) was selected, whose balance of properties (high degree of conversion coupled with impressive *T*_g_) looked particularly interesting as a model to study (Figure 2b). The simulation approach has been used by our group with some success with other high performance polymers such as epoxy resins [13] and cyanate esters [14] and both commercial and novel polybenzoxazines [15]. However, this has not previously been reported for telechelic species as the length of backbone confers an additional level of complexity to both the structure and the calculations.

## Methods

The molecular modelling program Accelrys Materials Studio version 5.5 [16] was utilised within this work and all the modelling work was carried out using an in house PC (Dell Optiplex 780, Inter Core Duo 3.00 Ghz, 4.00 GB RAM). The potential energy for all models throughout this work was calculated using the Condensed-phase Optimised Molecular Potential for Atomistic Simulation Studies (COMPASS) [17], a force field specifically designed for polymer calculations.

### Modelling the Thermoplastic Oligomer

**Figure 3:**
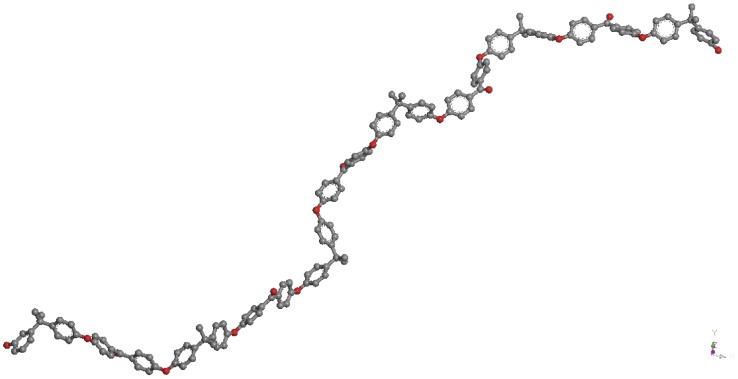
Simulated computational model in Accelrys Materials Studio of the single α,ω-dihydroxy terminated PEK oligomer (molecular weight 2200 Da, n = 5).

To model the precursor oligomer based on the low molecular weight poly(ether ketone) (PEK) (Figure 2a), the target density for the cell was set at 1.30 g cm^−3^, consistent with the density found for PEK (1.36–1.62 g cm^−3^) in the literature [18]. The Polymer Builder module was used to create a model containing 12 chains of the PEK each of 5 Monomers in length (Figure 3) and the Amorphous Builder module was then used to create a simulation cell of the target density. The cell was energy minimised until convergence was achieved (the convergence criterion was 0.001 kJ/mol difference between successive minimisations). The cell was replicated in 3 three dimensions by using periodic boundary conditions in the software to represent an effectively infinite chain. The inclusion of more than one chain in the simulation cell was used to allow for intermolecular interactions between the PEK chains and also to allow the modification of these chains by benzoxazine endcapping subsequently.

### Modelling the Telechelic Polybenzoxazine Thermoset Network

The Amorphous Cell module within Materials Studio was used to construct an amorphous cell with 12 telechelic precursors with the structural repeat unit shown in Figure 1 for a PEK backbone of length *n* = 5. To form the cross-linked polymer network (the polybenzoxazine) to different selected degrees of conversion, a curing programme produced in house [19] was employed with the COMPASS force field. For the construction of the network, the cut-off was set at 5.0 Å, the dynamics duration was set at 10,000 fs and the simulated cure temperature was 550 K.

### Modelling the Glass Transition and Thermal Degradation Temperatures for the Thermoset

The temperature ramped MD simulations were performed using the Temperature Cycle option in the Amorphous Cell Protocols. A collection of MD simulations was run over different temperatures, with decrements of 10 K from the starting temperature. The starting temperature was set at 600 K, and a total of 31 MD simulations were performed, ranging between 600 K and 300 K. At each temperature stage a 125 ps MD simulation was created. The first 25 ps of each simulation were used to equilibrate the system and the subsequent 100 ps simulation was used to record the results. In order to confirm that 100 ps was a sufficient timescale to equilibrate the volume of the cell, volume, energy and non-boned energy were plotted with respect to simulation time. This showed that all three parameters converged after 25 ps and remained stable for the duration of the simulation. This does not of course prove that there might be a transition to another stable minimum after this time period but for the purposes of this simulation it was sufficient. The NPT ensemble (298 K, 0.0001 GPa) with a time step of 1 fs was utilized with the Anderson thermostat in combination with the Parinello Barostat [20]. The NPT ensemble is used as it holds Pressure and temperature constant and allows the volume to change. As we monitor the change in volume with respect to temperature to observe the T_g_ then this ensemble serves this purpose quite well. We have carried out experiments using NVT for several polymeric systems and observed the change in pressure but this deserves fuller treatment in another publication. COMPASS was used with the atomic van der Waals summation, a cut-off at 10.0 Å, a spline width of 3.00 Å and a buffer width of 1.0 Å. The T_g_ is a second order phase change, which shows a change in thermal expansion coefficient when the temperature and volume of a polymer are plotted [21]. The point of gradient change in the plot pinpoints the position of the T_g_. The process of indicating the best point of gradient change can be quite complex. Hall *et al.* [22] developed an in-house technique of calculating this hinge point by finding when the fit quality of a line is at its maximum. This method is based on finding the best fit for a gradient change as a function of temperature. As the degradation temperature of polymers shows a similar change in volume as a function of the temperature, the same technique has been applied within this work.

### Modelling the Mechanical Properties

Mechanical properties were calculated using the full trajectory from the MD run in the Discover module, using Analysis, then Mechanical, then static mechanical properties calculation. Several determinations of moduli were taken starting from different initial configurations of the polymer model. Models were energy minimised with a default setting of 50000 steps and the majority of the models finished minimising at around 20000 steps. Mechanical properties were calculated as the average of three separate determinations.

## Results and Discussion

### Poly(ether ketone) PEK Precursor and Telechelic Benzoxazine Monomers

**Figure 4:**
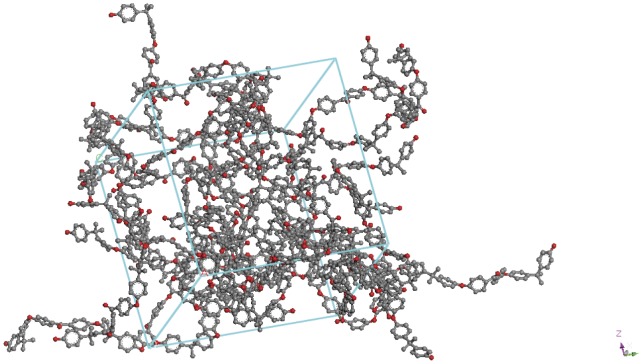
Simulated computational model in Accelrys Materials Studio of 12 chains of PEK (each n = 5) in PBC following energy minimisation.

In the original publication, the precursor was deliberately prepared to be an *α*,*ω*-dihydroxy terminated telechelic monomer by maintaining the stoichiometry of the reaction mixture (bisphenol A and 4,4′-dichlorobenzophenone, in the molar ratio 3∶2). Prior to constructing the network, the foundations were laid by preparing *in silico* the same precursor oligomer (Figure 2a and Figure 3). The extension of this model to a 12-chain simulation (comprising *ca.* 4000 atoms under periodic boundary conditions, PBC) (Figure 4) was undertaken to determine the effect of introducing more benzoxazine endcaps into the system. Figure 4 shows the PEK model in connected chain mode, where complete chains are connected to show the conformation of the main chain. The actual model is contained within the periodic cell (also shown in the figure) but would show disconnected chains, as the connections happen through the periodic cells. The ball and stick representation shows the different atom types (black for carbon, white for hydrogen and red for oxygen), giving a clear idea of the distribution of the atoms.

### Simulation of the Glass Transition Temperature and Decomposition Behaviour

**Figure 5:**
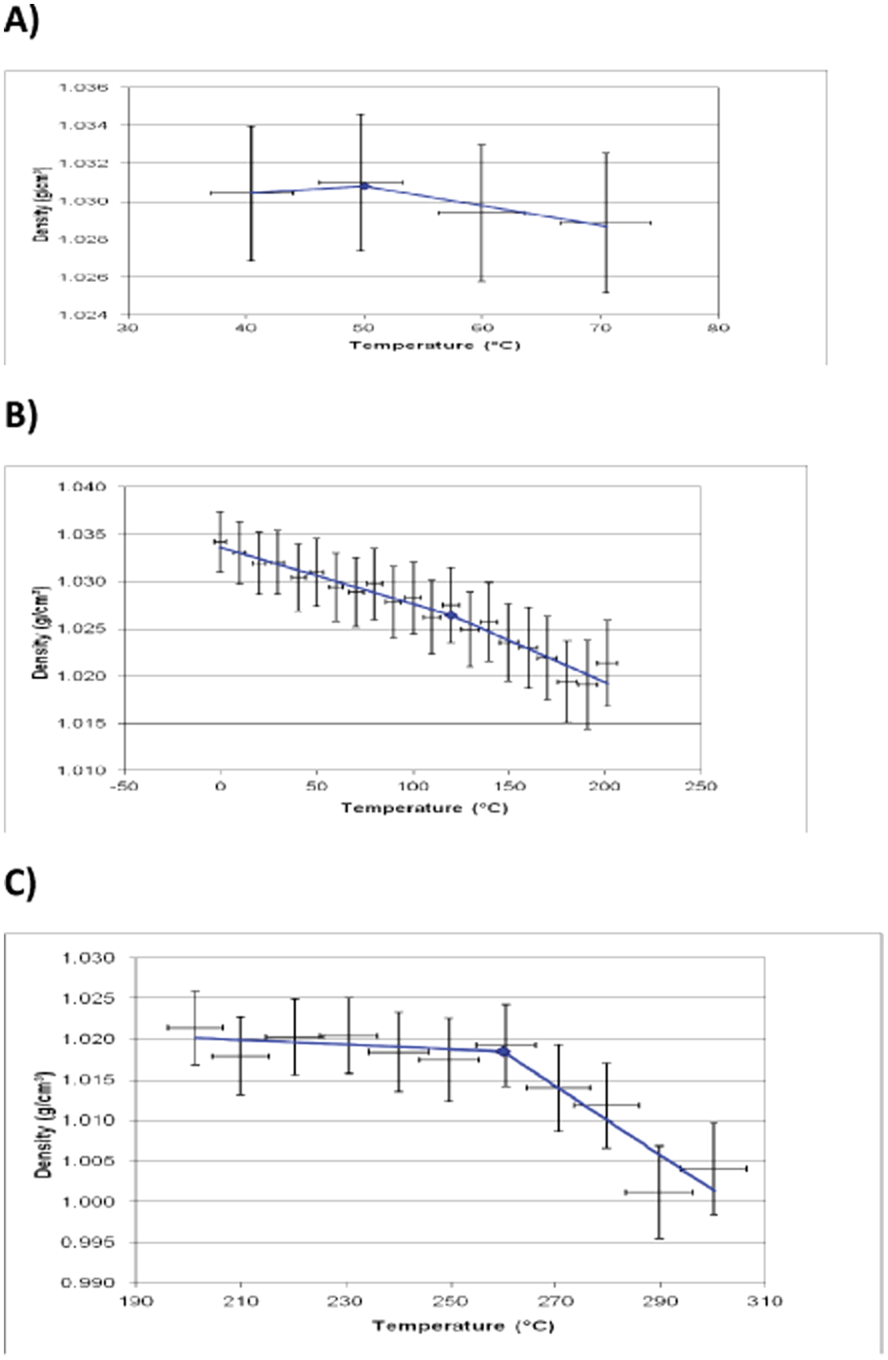
Plot of simulated density versus temperature from MD data for PEK oligomer 0-0. A) data from 30°C to 80°C showing point of β transition, B) data from −50°C to 200°C showing point of T_g_ and C) data from 190°C to 310°C showing point of T_d_.

An initial study was performed on the PEK oligomer (the precursor) for which some literature is available to allow comparison to be made. In this paper, we have adopted the following convention: each model is designated by two numbers, the first denoting the % of the chains endcapped and the second denoting the % of those benzoxazine rings that have been reacted to form crosslinks. Thus, the model 53-21 comprises a 3-D network based on oligomers (n = 5) in which 53% of the chains bear benzoxazine rings (the remainder are hydroxyl terminated) of which 21% have been reacted to form crosslinks. The PEK precursor is thus 0-0, *i.e.* no benzoxazine rings and no crosslinks. The temperature ramped MD study was performed on the model shown in Figure 4 comprising 12 pentamer (n = 5) chains of PEK, the results from the 100 ps data acquisition stage were recorded. For each simulation the batch average and standard deviation for both the temperature and the density were saved and are displayed in Figure 5. The graphs show simulated density versus temperature, each point on the graph is a single experiment where the information derived is the equilibrated density arrived at by the system after the molecular dynamics simulation. 100 ps was chosen for the dynamics simulation time as the density had stabilised in all cases after this time period. When we are dealing with molecular dynamics equilibration, one never knows if an additional period of simulation might not lead to a different density, so one tends to treat every simulation under the same conditions. When a polymer chain undergoes a thermal transition there is a change in the density of polymer at this temperature, e.g. when main chain motion takes place at the T_g_ then a drop in density is noted. This is because the volume of the system changes and as density is inversely related to volume, then an increase in volume relates to a decrease in density. As the T_g_ is a second order thermodynamic event, it does not have a defined experimental value and a range is often quoted. In the simulation we look to observe a change in density at the point where main chain motion takes place. In the past this was done by inspection of the data but this can be subject to interpretation, so a new method was developed that was less subject to interpretation. The axis on the right hand side of the graph is the transition mid-point certainty, which is calculated from fitting an ellipse to each point, thereby taking into account the uncertainty in both density and temperature and effectively finds the point of maximum deviation in the data. The red line on the graph represents these data. The dataset can be sub-divided to locate the deviations corresponding to the α transition, the T_g_ and the T_d_. The method employed is described in greater detail in reference 22.

Several distinct transitions, corresponding to reductions in density, are visible in the graph. There is a gradual drop in density (1.03 - 1.02 g cm^−3^) at around 50–170°C where there is a marked change in the gradient of the plot, which may correspond to the glass transition temperature (reported to be 163°C for the fully polymerised PEK [23]). The empirical measurement of T_g_ carried out by Ishida's group at Case Western Reserve University for the unfunctionalised oligomeric PEK was found to be around 127 to 147°C using DMA [5]. This marked drop in T_g_ is to be expected as this system is not fully polymerised. This event precedes another linear portion of the graph (120–260°C), which precedes a more marked drop in density, which appears to correspond to the degradation temperature of 0-0. Again there is a change in the slope in reference 5 at 260°C (a melting temperature of 361°C has been reported for the fully polymerised PEK [23]). Again it is to be expected that the degradation temperature of the oligomer would be markedly lower than the fully reacted polymer. Tsai *et al.* studied the first stage of decomposition of PEK (with a similar backbone structure) using pyrolysis GC/MS and identified 1,4–diphenoxybenzene and 4-phenoxyphenol as products at 450°C [24], indicating that the degradation of PEK is initiated by cleavage at chain ends and branches. Having validated the model for the thermoplastic polymer, the work was extended to address the properties of the crosslinked polybenzoxazine network.

**Figure 6:**
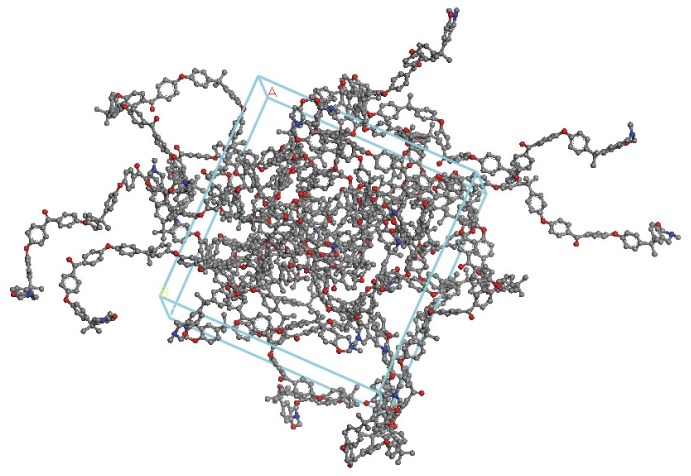
Simulated computational model in Accelrys Materials Studio of 12 chains of telechelic Polybenzoxazine in Periodic Boundary Conditions (PBC) following energy minimisation.

A similar construction method was initially adopted (*i.e.* formation of oligomers such as the sample comprising 12 chains of uncrosslinked *bis*-benzoxazine telechelics shown in Figure 6) and the results of the molecular mechanics (MM) study yielded bond lengths and angles that agreed well with experimental data (Table S1). Examination of the MM data confirms that the configuration of the polymers have remained largely unchanged in the bulk model, although the bulk density becomes significantly reduced as the degree of endcapping is increased (Table 1 and Figure 6). The nitrogen atoms are shown in blue in Figure 6 and the benzoxazine groups can be clearly seen. Fortunately, empirical density data were available for similar benzoxazines allowing comparison [25]. Several samples were modelled (53-14, 53-21, 67-31, 83-35, 92-40, 100-45, 100-54) to explore the effect of the efficiency of the endcapping reaction and the degree of conversion on the mechanical properties.

**Table 1:** Degree of benzoxazine endcapping used in the Computational Models with resulting densities [28], [29].

Benzoxazine endcapping (%)	0	41.67	58.33	66.67	83.33	91.67	100
Density (g/cm^3^)	1.30	1.27	1.24	1.23	1.22	1.21	1.20

**Figure 7:**
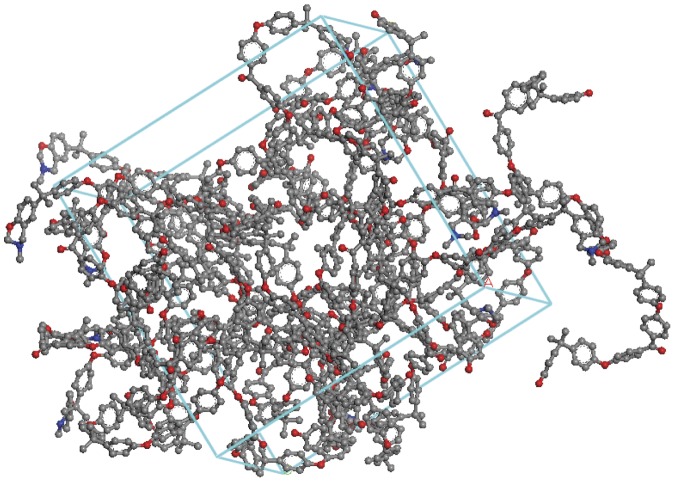
Simulated computational model in Accelrys Materials Studio of crosslinked polybenzoxazine (53-21) cured to 21% conversion in PBC following energy minimisation.

**Figure 8:**
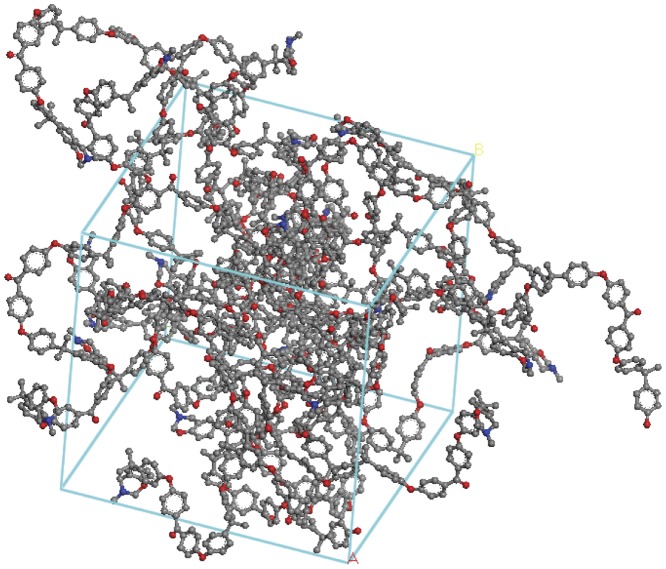
Simulated computational model in Accelrys Materials Studio of crosslinked polybenzoxazine (92-40) cured to 40% conversion in PBC following energy minimisation.

**Figure 9:**
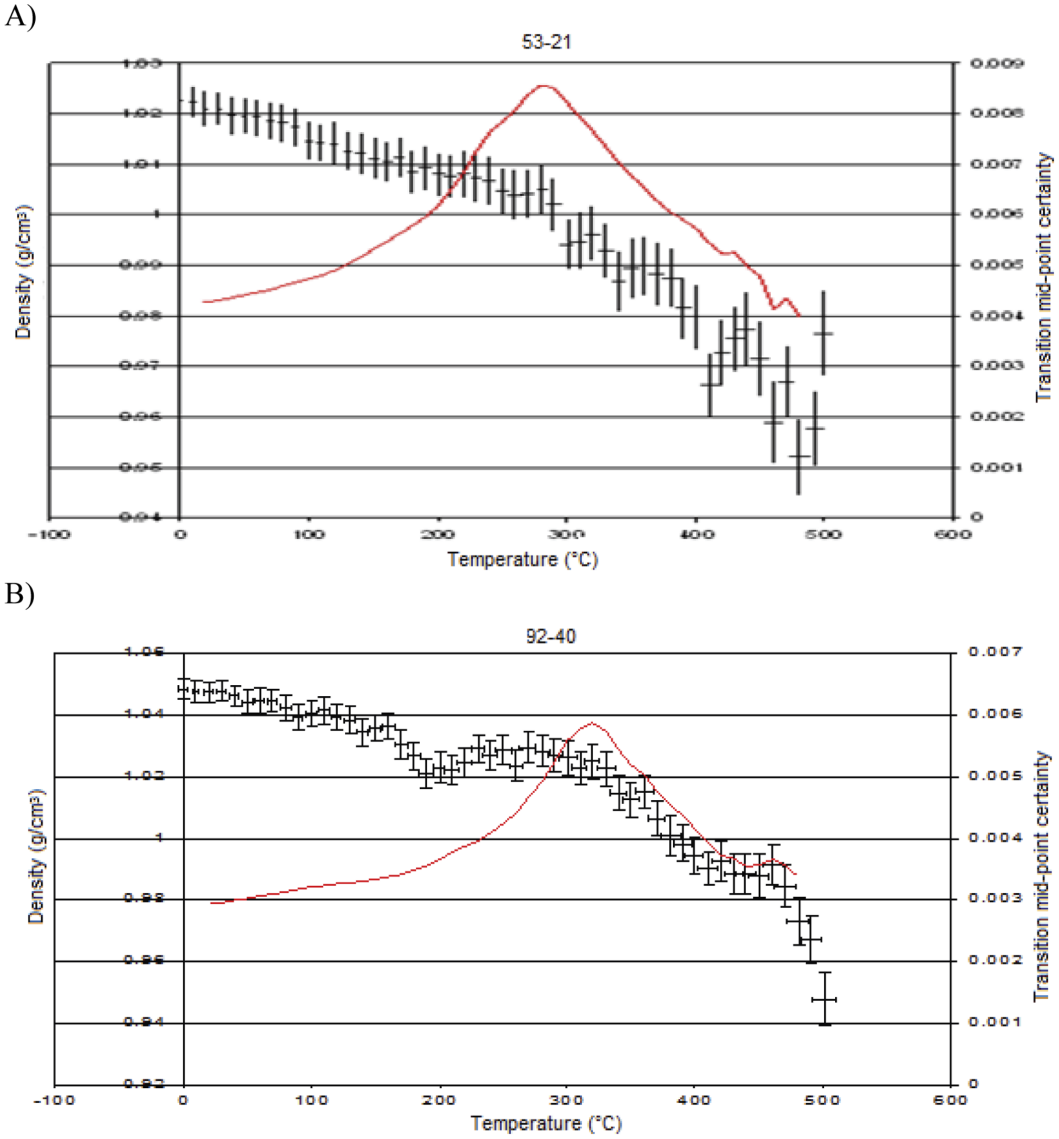
Simulated density of crosslinked polybenzoxazines (a) (53-21) and (b) (92-40) *versus* temperature. The red line represents the transition mid point certainty (right hand side axis) and locates in this case the T_d_.

Two examples of the crosslinked networks are shown (Figures 7 and 8) to demonstrate the effect of crosslink density upon the volume that the cell occupies. It can be seen in these figures that as the crosslinking increases the calculated density of the cells also increases. A comparison of Figures 7 and 8 shows the more dense nature of the models. Having prepared each network structure *in silico* using the in house construction programme, at each temperature stage in the temperature ramped MD simulation, the results from the 100 ps data acquisition stage were recorded. For each simulation the batch average and standard deviation for both the temperature and the density were saved and representative data are displayed in Figure 9. In each case the different temperature transitions (α, β, T_d_) were noted and are displayed in Table 2 (where T_d_ represents the onset of thermal degradation).

**Table 2:** Predicted thermal transitions for PEK samples with % of benzoxazine endcapped - % of crosslink for each of the models.

Oligomer models	β (°C)	*T*_g_ (°C)	*T*_d_ (°C)
0-0	50	120	260
53-14	50	140	300
53-21	80	220	300
67-31	50	220	350
83-35	70	230	330
92-40	80	180	310
100-45	120	270	400
100-54	120	280	400

When examining the thermal degradation behaviour (T_d_) conventional MD simulations use harmonic potentials for bond lengths and tend to operate well when bond lengths only vary between about 75% of their equilibrium values. This means that they cannot be used to model bond breaking scenarios. Reactive molecular dynamics *e.g.* in REAXFF [26] replaces these harmonic potentials with bond order calculations (amongst other changes) and this does allow bond breaking and formation. This technique is currently finding wider application in the literature but it is the object of this work to pursue the limits of conventional molecular dynamics. In this investigation the predicted thermal degradation temperatures of the oligomers will be compared to the reported empirical data for the synthesised materials in order to evaluate whether computational models can be used to accurately predict the degradation temperatures of these materials.

The thermal decomposition of polybenzoxazines involves a three-step process that includes the cleavage of the hydrocarbon backbone, the breakdown of the oxazine rings and a decomposition of the solid residue to form a char [27]. As the computational model of atomic bonds used in this simulation are simple harmonic oscillators, atoms within the computational model are not able to dissociate. Therefore, the thermal decomposition of a molecule cannot be replicated by conventional computational studies. However, conventional computational models are still able to predict the degradation temperatures of polymers. This is because at high temperatures the weak forces holding the polymer chains together are overcome by the thermal energy and the chains can move apart. This is conversely restricted by the degree to which the chains are covalently bonded together, i.e. by the cross links. Based on the empirical study [5], the telechelic PEK capped with benzoxazine started to degrade at 450°C (from TGA measurements) and this was mainly attributed to the degradation of the main chain itself. The temperature for 5% weight reduction was 478°C experimentally. In the *in silico* study, the simulated degradation temperature (Table 2) vary from 300–400°C for systems varying from 14% crosslinked to 54% crosslinked. Clearly the simulated degradation temperature rises with the number of crosslinks in the system and this is to be expected as a more highly crosslinked system would be consequently harder to degrade. We do not have an actual experimental value for the cross link density in the experimental system but benzoxazines are in practice never less than 70% crosslinked. Hence the simulated value of 400°C for the 54% crosslinked system is encouraging and points to the experimental system being more highly crosslinked than this model.. The influence of crosslinking on the thermal stability is clearly evident.

### Simulation of Mechanical Properties for Telechelic Polybenzoxazines

The simulated mechanical properties are shown in Table 3. Different starting models were taken in all cases so the results in each case reflect the variation in the starting conformations of the models. Therefore an average of the three determinations of the mechanical properties was made.. The result for the unmodified PEK (0-0 in Table 3) shows a tensile modulus of 4.76 GPa, the experimental value is 3.6 Gpa. This value is not unreasonable as we are dealing with relatively simple models without any defects and would therefore expect an overestimate. Similarly the Poisson's ratio is 0.25, compared to the experimental value of 0.3. The simulated results for the benzoxazine modified molecules show a consistent increase in the Young's modulus above that of the unmodified PEK in line with the increasing stiffness caused by the introduction of crosslinking. As we are modifying two variables at the same time, i.e. the number of benzoxazine groups endcapped along with the amount of crosslinking, we would not expect a linear increase anyway. There is always the question of how representative the models are of the ‘real’ situation and there is still great debate about the difficulty of optimising chains of this length with molecular mechanics. Taking this into account, it is clear that the introduction of benzoxazine endcapping and crosslinking increases the tensile modulus, bulk modulus and shear modulus. Hence there is a predicted increase in the mechanical properties over the inherently ‘tough’ starting material. Unfortunately, the experimental mechanical properties of the telechelic benzoxazines have not yet been determined, so this is a testable prediction of the expected values.

**Table 3:** Simulated Mechanical properties of the models.

Models	Tensile Modulus	Poisson's Ratio	Bulk Modulus	Shear Modulus	Lamé	Lamé
	(GPa)		(GPa)	(GPa)	(λ)	(μ)
0-0	4.76	0.25	3.74	1.94	2.44	1.94
53-14	9.89	0.27	7.29	3.9	4.69	3.9
53-21	10.27	0.31	8.98	3.92	6.37	3.92
67-31	7.32	0.26	5.4	2.92	3.45	2.92
83-25	7.74	0.31	6.79	2.95	4.82	2.95
92-40	5.57	0.26	4.48	2.27	2.97	2.27
100-45	8.19	0.24	5.29	3.31	3.08	3.31
100-54	8.39	0.31	7.4	3.2	5.26	3.21

## Conclusions

A series of benzoxazine end capped PEK polymers were constructed by molecular modelling and their thermal and mechanical properties simulated. It has been possible to predict the β tranistion, T_g_ and T_d_ for the entire series of polymers. The simulated data for the starting material yielded values of 50, 120 and 260°C, which compare well with the experimental data that has been determined for this system [5]. The addition of the benzoxazine endcapping and crosslinking serves to increase the temperature of all the thermal events, eventually rising above those of the unfunctionalised and fully polymerised PEK starting material. The fully polymerised PEK has a Tg of 163°C and Tm of 366°C and our model of 100%-54% has values of 280°C and 400°C. The simulated mechanical properties show a similar trend with those of the starting material, i.e. the tensile modulus is approximately doubled by the modification. This study has shown that molecular dynamic simulations can predict physical and mechanical data for polybenzoxazines derived from telechelic monomers as well as providing molecular insight into the structures formed by these systems.

## Supporting Information

Table S1 **Selected Bond lengths of selected bond types in the PEK telechelic obtained using Materials Studio (for a single chain and twelve chains) at room temperature (for the given configurations).**
(DOC)
