# Supplementary material for: Prediction of Selected Physical and Mechanical Properties of a Telechelic Polybenzoxazine by Molecular Simulation
Source: PLoS One. 2013 Apr 8;8(4):e61179. doi: 10.1371/journal.pone.0061179 (PMC3620114; doi:10.1371/journal.pone.0061179)
Supplement: Table S1 — Selected Bond lengths of selected bond types in the PEK telechelic obtained using Materials Studio (for a single chain and twelve chains) at room temperature (for the given configurations). (DOC) [file pone.0061179.s001.doc]

**Table S1 Selected Bond lengths of selected bond types in the PEK telechelic obtained using Materials Studio (for a single chain and twelve chains) at room temperature (for the given configurations)**

|  | | | | | | |
| --- | --- | --- | --- | --- | --- | --- |
| **Bond lengths (Ǻ)** | | | | **Bond angles (˚)** | | |
| **Single Chain Model** | | | | | | |
|  | **Modelling**  **data** | **Empirical data^6^** |  | | **Modelling**  **data** | **Empirical**  **data^6^** |
| C9-O | 1.38 | 1.36 | C9-O-C3 | | 114.9 | 119.9 |
| O-C3 | 1.43 | 1.45 | O-C3-N | | 112.0 | 113.5 |
| C3-N | 1.46 | 1.43 | C3-N-C2 | | 111.9 | 107.9 |
| N-C2 | 1.46 | 1.47 | N-C2-C4 | | 107.3 | 110.2 |
| C2-C4 | 1.51 | 1.51 | C2-C4-C9 | | 120.6 | 117.7 |
| N-C1 | 1.46 | - | C4-C9-O | | 122.6 | 123.1 |
| C9-C4 | 1.40 | 1.39 | C3-N-C1 | | 114.3 | - |
| **12-Chain Model** | | | | | | |
| C9-O | 1.38 | 1.36 | C9-O-C3 | | 117.9 | 119.9 |
| O-C3 | 1.43 | 1.45 | O-C3-N | | 112.0 | 113.5 |
| C3-N | 1.46 | 1.43 | C3-N-C2 | | 111.9 | 107.9 |
| N-C2 | 1.47 | 1.47 | N-C2-C4 | | 107.3 | 110.2 |
| C2-C4 | 1.51 | 1.51 | C2-C4-C9 | | 120.6 | 117.7 |
| N-C1 | 1.46 | - | C4-C9-O | | 122.6 | 123.1 |
